# Evidence for Female-Biased Dispersal in the Protandrous Hermaphroditic Asian Seabass, *Lates calcarifer*

**DOI:** 10.1371/journal.pone.0037976

**Published:** 2012-06-12

**Authors:** Gen Hua Yue, Jun Hong Xia, Feng Liu, Grace Lin

**Affiliations:** Molecular Population Genetics Group, Temasek Life Sciences Laboratory, National University of Singapore, Singapore, Republic of Singapore; Institut Pluridisciplinaire Hubert Curien, France

## Abstract

Movement of individuals influences individual reproductive success, fitness, genetic diversity and relationships among individuals within populations and gene exchange among populations. Competition between males or females for mating opportunities and/or local resources predicts a female bias in taxa with monogamous mating systems and a male-biased dispersal in polygynous species. In birds and mammals, the patterns of dispersal between sexes are well explored, while dispersal patterns in protandrous hermaphroditic fish species have not been studied. We collected 549 adult individuals of Asian seabass (*Lates calcarifer*) from four locations in the South China Sea. To assess the difference in patterns of dispersal between sexes, we genotyped all individuals with 18 microsatellites. Significant genetic differentiation was detected among and within sampling locations. The parameters of population structure (*F*_ST_), relatedness (*r*) and the mean assignment index (mAIC), in combination with data on tagging-recapture, supplied strong evidences for female-biased dispersal in the Asian seabass. This result contradicts our initial hypothesis of no sex difference in dispersal. We suggest that inbreeding avoidance of females, female mate choice under the condition of low mate competition among males, and male resource competition create a female-biased dispersal. The bigger body size of females may be a cause of the female-biased movement. Studies of dispersal using data from DNA markers and tagging-recapture in hermaphroditic fish species could enhance our understanding of patterns of dispersal in fish.

## Introduction

Dispersal is an important life history trait. It influences individual fitness, reproductive success, genetic variation and relationships among individuals within populations, gene flow among populations and the potential to colonize in new habitats [1]. The study of dispersal has been an active research area in evolutionary biology and molecular ecology [2]. In the early 1980s, studies on sex-biased dispersal in both birds and mammals set the basis for subsequent researches on theories of sex-biased dispersal [3]. Generally, mammals display male-biased dispersal [4], whereas most of the birds show the reverse pattern [5], [6].

Several theories have been developed to explain the sex-biased dispersal. They relate the difference in dispersal between sexes to gender-specific differences in the advantages that philopatry conveys to males and females, or to sex-specific effects of fecundity costs. Competition for mates and breeding resources, and inbreeding avoidance can result in sex-biased dispersal [3], [5]. The sex that gains more from prior ownership of a territory would be favoured by selection to move less when the fitness effects of gaining and defending reproductive resources are different between two sexes [3], [5], [7]. Besides resource competition, sex-biased dispersal was also attributed to mating systems [8], [9]. Competition among kin and inbreeding avoidance can also produce sex-biased dispersal in mammals (Handley & Perrin 2007), birds [6], [10] and some fish species [11], [12], because competition among kin diminishes the inclusive fitness. Whether avoidance of kin competition can result in sex-biased dispersal is associated with the possibility that the two sexes compete for the same resources [13]. In polygamous mating systems, the breeding success of males may be limited mainly by the number of mating partners (i.e. females). Therefore, the local competition for mating with females is possibly higher for males. In contrast, when males make no parental investment or the male contribution to parental care is minor, the competition for local resource among relatives for limited breeding resources may be greater among females [14], [15]. Previous studies suggested that the sex differences in migrating patterns are associated with the balance among kin for mates and/or for resources [16], [17], [18]. When the breeding resources limit the female reproductive success in polygynous or promiscuous systems, or when the effect of local competition among males and females is the same in monogamous systems, dispersal is not sex-biased [19].

Sex-biased dispersal in fish has been studied only in a few species, although there are over 30,000 teleost fish species on the earth [20]. Male-biased dispersal has been reported in some fish species, such as brook trout [11] and three-spined stickleback [21]. Female-biased movement has also been described in Lake Malawi cichlids [22], dwelling Dolly Varden [23] and salmonids [24]. In salmonids, migrating individuals moved to beneficial habitats to feed and gain larger body size [24]. It is believed that female-biased dispersal may have caused the female-biased sexual size dimorphisms in some fish species [25], [26], [27]. The patterns and evolutionary significance of sex-biased dispersal in protandrous hermaphroditic species remain unexplored. The genetic differentiation of most marine fish populations is usually weak [28] which is probably the consequence of unrestricted gene flow in marine environment [28], [29]. Therefore, it is believed that dispersal is unbiased between sexes in these marine fish species where population differentiation is low. In fishes, direct observation of movements is very difficult, but dispersal can be inferred using polymorphic DNA markers, such as microsatellites and other DNA markers [12], [21].

The Asian seabass (also called barramundi), *Lates calcarifer*, belongs to the family Centropomidae. It is distributed in coastal waters, freshwaters and estuaries through the whole of Southeast Asia to Papua New Guinea and northern Australia. It can also be found from western India to the Bay of Bengal [20]. It has a complex life history and is a protandrous hermaphrodite, which starts life as male first and later changes to female after males reached sexual mature at the age of 3 to 4 years [30]. The transition from male to female is short (i.e. a few months) and may not occur in all individuals [31]. The fecundity of *L. calcarifer* is among the highest of any teleost fish, and its life span is over 25 years. Usually, an adult female produces 0.5–40.0 million eggs [32], [33]. Spawning takes place in the sea all year around in Southeast Asia, but only occurs in the sea from October to February in Australia [30], [32], [33]. Females spawn every three months in Southeast Asia while males reproduce all year around [34]. *Lates calcarifer* is a non-guarding species. There is no parental involvement in the development of fry and juvenile fish [30], [32], [33]. *Lates calcarifer* is catadromous and migrates substantially to specific spawning grounds in order to breed [32], [35], [36]. Previous data on tagging and recapture showed that juvenile *L. calcarifer* remained resident until reaching sexual maturity at the age of 3–4 years [37], [38] and during spawning, sexually matured individuals migrated to coastal areas for breeding [38]. Recent studies indicated that adult *L. calcarifer* did not always migrate to breeding grounds to spawn, with a lifetime non-participation rate of as much as 50% and bigger individuals migrated more than smaller ones [39], [40]. Because *L. calcarifer* is a broadcast breeder, there might be little interaction between males and females. Since species with external fertilization and little or no post-spawning parental investment may not exhibit sex-biased movement [5], [41], we hypothesize that dispersal of *L. calcarifer* is unbiased between the two sexes.

In this study, we collected 549 Asian seabass individuals from four locations along the cost of Thailand, Malaysia, Singapore and Indonesia. To quantify genetic parameters in these four sampling locations, we used 18 polymorphic microsatellites to genotype the 549 individuals. We tested the hypotheses of unbiased dispersal in *L. calcarifer* by combining genetic parameters estimated in this study with data on tagging-recapture published by Australian scientists in the past 30 years [35], [36], [38], [42]. To our best knowledge, this is the first study aiming to analyze the dispersal patterns and the potential reasons for sex-biased dispersal in a protandrous hermaphroditic marine fish species. The results of this study could add new data on dispersal patterns of fish species, in which the patterns of dispersal have not been extensively studied, thus enhancing our understanding of dispersal patterns of fish.

## Materials and Methods

### Ethics Statement

All handling of fishes was conducted in accordance with the guidelines on the care and use of animals for scientific purposes set up by the Institutional Animal Care and Use Committee (IACUC) of the Temasek Life Sciences Laboratory, Singapore. The IACUC has specially approved this study within the project “Molecular Breeding of Asian seabass” (approval number is TLL (F)-003-09).

### Sampling

**Figure 1:**
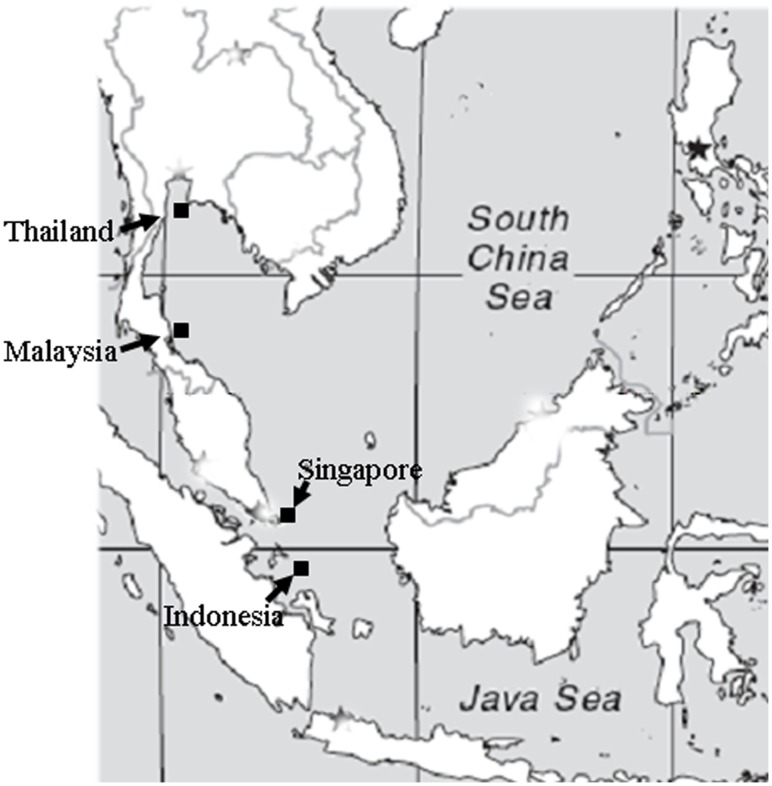
Map of sampling locations of Asian seabass along the coast of Thailand, Malaysia, Singapore and Indonesia.

We collected 549 adult (3–4 years old) *L. calcarifer* individuals from four locations in South China Sea along the coast of Thailand, Malaysia, Singapore and Indonesia (Table 1; Fig. 1). We brought all fish back to fish facility in Marine Aquaculture Centre, Singapore. We determined the age of *L. calcarifer* by counting growth rings on their scales (otoliths) according to McDougall [43]. All individuals were weighted and sexed by squeezing for sperm or eggs. We collected fin clips from each fish and stored them individually in 95% ethanol till DNA extraction.

**Table 1:** Samples of Asian seabass used in this study.

Sampling location	N	F	M	F BW (kg) TBL (cm)	M BW (kg) TBL (cm)	*H*_E_	*A*_R_	*F*_IS_
Malaysia	165	105	60	4.82±1.28* 68.4±0.38*	3.05±0.68 52.6±0.40	0.702	8.23	0.069
Singapore	104	53	51	4.38±0.17* 65.5±0.76*	2.96±0.45 53.1±0.55	0.681	7.81	0.008
Thailand	132	76	56	4.05±1.06* 66.2±0.57*	2.56±0.27 51.9±0.27	0.713	8.92	0.073
Indonesia	148	82	66	5.20±0.92* 72.9±0.48*	3.10±0.39 62.9±0.39	0.697	8.77	0.027

N, number of sampled individuals; M, males; F, females; BW, average body weight ± SD; TBL, total body length; *H*_E_, gene diversity; *A*_R_, allelic richness; *F*_IS_, inbreeding coefficient and *, *P*<0.05 indicating significant difference between males and females.

### DNA Isolation and Microsatellite Genotyping

We extracted total DNA from fin clips of each fish using a method developed by us [44]. Extracted DNA was eluted in distilled water. DNA quality and quantity were examined using electrophoresis on 1% agarose gels and Nanodrop (Thermo Scientific), respectively.

We genotyped all individuals using 18 polymorphic microsatellite markers (see details in Table 2) developed by us previously [45], [46], [47] due to their polymorphism, the ease of PCR amplification and scoring genotypes. One primer of each pair was labelled with a fluorescent dye (either FAM or Hex). PCR was performed in PTC-100 thermal-cyclers (MJ Research) using the following PCR program: 94°C for 3 min, followed by 36 cycles of 94°C for 30 s; 55°C for 30 s; 72°C for 30 s and a final extension at 72°C for 10 min. Twenty-five µl reaction mixes consisted of 20 ng of DNA, 0.5 µm of each primer, 200 µm of each dNTP, 0.5 units of Taq-polymerase (Finnzymes) and 1×reaction buffer with 1.5 mm MgCl_2_. PCR products were resolved on ABI3730×l automated sequencers (Applied Biosystems). GENEMAPPER v.4.1 (Applied Biosystems) was used to score fragments.

**Table 2:** Details of the 18 microsatellite loci of Asian seabass used in this study.

Locus	GenBank Accession no.	LG	*A*	*H*_O_	*H*_E_	*F*_IS_	*F*_ST_
*Lca002*	AF007943	10	9	0.672	0.703	0.043	0.021
*BMS08*	AF404076	–	4	0.459	0.499	0.080	0.021
*Lca016*	AF406080	–	15	0.778	0.822	0.053	0.017
*Lca020*	AF404082	16	11	0.723	0.750	0.036	0.007
*Lca021*	AF404083	17	7	0.809	0.823	0.017	0.006
*Lca040*	AF404099	22	8	0.687	0.699	0.018	0.018
*Lca050*	AY998845	20	2	0.453	0.455	0.003	0.015
*Lca057*	AY998849	17	10	0.725	0.736	0.015	0.022
*Lca058*	AY998850	11	16	0.811	0.891	0.090	0.019
*Lca062*	AY998854	6	14	0.766	0.882	0.132	0.020
*Lca063*	AY998855	13	10	0.641	0.705	0.092	0.036
*Lca064*	AY998856	2	10	0.707	0.783	0.098	0.015
*Lca069*	AY998859	15	6	0.719	0.775	0.071	0.024
*Lca070*	AY998860	14	8	0.698	0.745	0.063	0.025
*Lca072*	AY998861	–	2	0.203	0.219	0.075	−0.001
*Lca074*	AY998863	12	10	0.610	0.676	0.097	0.018
*Lca086*	AY998873	8	23	0.909	0.901	−0.009	0.020
*Lca098*	AY998880	5	20	0.732	0.763	0.040	0.031
Average		–	10.28	0.712	0.666	0.065	0.022

LG, linkage group; *A*, number of alleles; *H*_O,_ observed heterozygosity; *H_E_*, expected heterozygosity; *F*_IS_, inbreeding coefficient; *F*_ST_, differentiation in allele frequencies; and -, not mapped to linkage groups.

### Data Analysis

Since the existence of null alleles can result in false homozygotes and generate a pattern similar to the Wahlund effect [48], we used software MICRO-CHECKER 2.2.3 [49] to infer null alleles, stuttering bands and allele dropout. We calculated allele frequencies, allele number, observed (*H*_O_) and expected heterozygosities (*H*_E_) for each population using software GDA [50] and allelic richness, a parameter reflecting allelic diversity independent of sample size using software FSTAT v.2.9.3 [51].

We conducted hierarchical analysis of molecular variance (AMOVA) and estimated *F*_ST_ among the sampling locations, based on allele frequency information [52] using the program ARLEQUIN version 3.1 [53]. We analyzed variance components among and within sampling locations. We examined the isolation by distance with the online program IBDWS [54]. The statistical significance of the correlation between the pairwise genetic distance [*F*_ST_/(1−*F*_ST_)] and the geographical distance (log of distance in kilometers between populations along the coast line), was obtained by the use of Mantel tests.

We also examined the fine-scale population genetic structure for all 549 individuals collected along the coast of Thailand, Malaysia, Singapore and Indonesia using the program STRUCTURE version 2.3.3 [55]. This program uses the genotypes of each individual to find the optimal population number (*K*) that minimizes Hardy-Weinberg and linkage disequilibria using a Bayesian clustering algorithm and without assigning individuals to populations as a priori [56]. In simulation, we used an admixture model with correlated allele frequencies. We performed 10 independent runs for *K*  = 1−20 using 100 000 burn-in steps and 100 000 Markov Chain Monte Carlo repetitions to ensure chain stabilization as suggested in the documentation for STRUCTURE software version 2.3.3 [55]. We chose the optimal *K* by identifying the mean maximum estimated logarithm of the probability of the data, Ln Pr (X/*K*), for each *K*, and computing the posterior probality of each *K* using formula given by Pritchard and Wen [55]. We also used the method of Evanno et al [57] to obtain the optimal *K*. To examine the proportion of males and females migrating to other locations, we also used *K*  = 4 for the LOCPRIOR model to cluster individuals by providing priors for the Bayesian assignment process based on the sampling locations. The LOCPRIOR model allows structure to be detected with lower levels of divergence and is not biased towards detecting structure when it is not present [58].

We examined the difference in dispersal between sexes using methods described by Goudet et al. [59] and software FSTAT v.2.9.3 [51]. We examined whether the genotypes at each locus deviated from Hardy–Weinberg equilibrium (HWE) within populations by calculating *F*_IS_. We analyzed population differentiation using *F*_ST_ [60] and determined the statistical significance (*P*) of the *F* indices by 10 000 randomizations as implemented in FSTAT v.2.9.3. To detect possible difference in dispersal between sexes, we quantified relatedness (*r*) [61], mean assignment index (mAIC), variance of the assignment index (vAIC), deviation from HWE (*F*_IS_) and differentiation among populations (*F*_ST_) for males and females separately over all four populations. The assignment index (AIC*)* is an estimate of the probability of a genotype belonging into a given population. Positive values of this index mean that the individual is a resident and negative values indicate that it is a migrant as AIC is centred on zero. In the case of sex-biased migration, the average AIC index (mAIC) for the sex that disperses is lower than that for the more philopatric sex because migrating individuals have lower AIC values than resident ones. Members of the dispersing sex include both residents and immigrants; therefore vAIC is larger for the dispersing sex. The *p* values of differences in these within-population indices were determined using the randomization method with 10,000 permutations using software FSTAT v.2.9.3. Since we used microsatellites to assess the dispersal patterns, we could only detect the short-term dispersal patterns as this signal disappears after the migrating individuals mate, due to the Mendelian segregation of biparental markers [59].

To examine whether there is difference of migration distance between adult males and females, we also retrieved and analyzed tagging and recapture records released by Infofish Services (http://www.info-fish.net/reporting_recapture.html). We only used the data of tagged adult fish, which were captured at least one year after tagging to ensure that fish had enough time to migrate. We compared the average migrating distance between male and female adult individuals using t-Test as implemented in software Microsoft Excel 2003. We also analyzed the relationship between migration distance and body length using the analysis tool “Regression” in Microsoft Excel 2003.

## Results

### Sex, and Body Weight and Length

We collected 549 individuals in four locations along the cost of Thailand, Malaysia, Indonesia and Singapore (Table 1 and Figure 1). All fishes used in this study were at the age of 3–4 years. In all 549 individuals, the sex ratio was female-biased (F:M  = 1.36). In each population, the sex ratio was also female-biased. The ratio of F:M ranged from 1.04 for the sampling location Singapore to 1.75 for the sampling location Malaysia. The average body weight and length of females were significantly (*P*<0.05) bigger than those of males in all four populations (Table 1).

### Microsatellite Polymorphism

All 549 individuals were successfully genotyped for each locus. The examination of genotypes in each locus with software MICRO-CHECKER revealed that there were no null alleles, no stuttering bands or allele dropout in all 18 loci. The average allele number/locus was 10.28. All loci conformed to HWE (Table 2).

### Population Differentiation

The overall differentiation among the four populations was low (*F*_ST_  = 0.022, *P*<0.05). The pairwise genetic differentiation was also low (*F*_ST_ ranged from 0.013 to 0.048; Table 3). But the genetic differentiation between pairwise populations was statistically significant (*P*<0.05). Statistical analysis with Mantel tests revealed that there was no isolation by distance relationships among the four populations (*r*  =  −0.16, *P*>0.05). The analysis of molecular variances (AMOVA) of microsatellites revealed that the variation within and among sampling locations was 97.76% (*P*<0.05) and 2.24% (*P*<0.05), respectively.

**Table 3:** Pairwise *F*_ST_ estimates between sampling locations of Asian seabass.

	Malaysia	Singapore	Thailand
Malaysia			
Singapore	**0.021**		
Thailand	**0.019**	**0.048**	
Indonesia	**0.013**	**0.019**	**0.015**

Estimates significantly higher than zero are in bold (*P*<0.05 in all cases).

**Figure 2:**
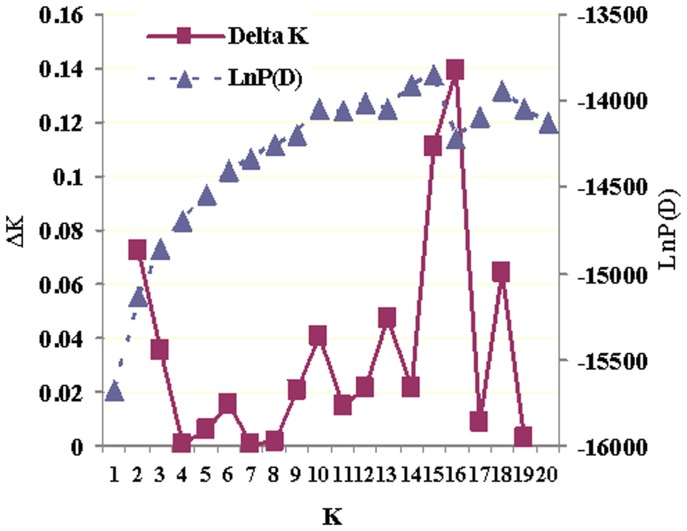
Results from the program STRUCTURE analysis of Asian seabass (*Lates calcarifer*, *n*  = 549) from four sampling locations along the coast of Thailand, Malaysia, Singapore and Indonesia. Plot displays mean log-likelihood LnP(D) and *ΔK* values for 10 independent runs for each value of K for K  = 1−20. The highest value was at K  = 15 and *ΔK*  = 15, indicating that the four sample locations likely form 15 populations.

Using the method described by Pritchard et al. [56], the optimal *K*-value revealed by STRUCTURE version 2.3.3 was *K*  = 15 (Figure 2), suggesting that the four sampling locations along the coast of Thailand, Malaysia, Singapore and Indonesia consist of 15 genetic clusters. Based on the *ΔK* method [57], the *ΔK* value maximized at *K*  = 15 (Figure 2), confirming that the 549 individuals collected the four locations could form 15 clusters. These data indicate population differentiation among the four sampling locations and sub-structuring within sampling locations. To examine the proportion of males and females migrating to other locations, we also used *K*  = 4 for the clustering analysis. The largest proportion (ranging from 31.1% for the location Indonesia to 51.0% for the location Singapore) of the individuals collected from a location was clustered into their original sampling locations (Table 4). Among the resident fish from each location, the proportion of males was much higher than that of females, while in the migrant individuals, the female proportion was much higher than the male proportion (Table 4).

**Table 4:** Proportion (%) of memberships of each pre-defined population of Asian seabass in each of four clusters inferred using software STRUCTURE.

Given population	Inferred clusters								Number of individuals
	1		2		3		4		
	M	F	M	F	M	F	M	F	
Malaysia	21.8	13.3	2.4	29.7	4.2	9.7	7.3	11.5	165
Singapore	5.8	11.5	36.5	14.4	3.8	7.7	4.8	15.4	104
Thailand	5.3	8.3	1.5	7.6	28.8	10.6	6.8	31.1	132
Indonesia	6.1	14.2	10.1	19.6	6.8	12.2	21.6	9.5	148

M: male and F: female.

### Sex-biased Dispersal

We estimated the genetic parameters separately for each sex using genotypes at all 18 microsatellites. The mean assignment index (mAIC) in females (−0.89) was significantly different from the mean assignment index in males (0.88, *P*  = 0.01, Table 5). Females showed higher variance in AIC values but the difference was not significant (female vAIC variance  = 30.37, male vAIC  = 29.26, *P*  = 0.76, Table 5). The population differentiation (*F*_ST,_ 0.036 for male vs 0.023 for female) and relatedness (*r*, 0.066 vs 0.044) were significantly (*P*  = 0.01) higher for males than for females (Table 5). The heterozygosity deficiency (*F*_IS_) was higher in males than that in females (0.046 vs. 0.035, *P*  = 0.036).

**Table 5:** *F*-statistics, relatedness (*r*), mean assignment (mAIC) and variance assignment (vAIC) for each sex in Asian seabass.

Sex	*F*_IS_	*F*_ST_	*r*	mAIC	vAIC
Males	0.046	0.036	0.066	0.88	29.26
Females	0.035	0.023	0.044	−0.89	30.37
*P*	0.039	0.010	0.010	0.010	0.760

Significance (*P*) was assessed using the randomisation method of Goudet *et al*. (2002).

We retrieved the records of tagging and recapture for 23 adult individuals released by Infofish Services. Analyzing the average migrating distance between male and female adults revealed that the females migrated more than males (75.7±23.1 km for 11 males vs 357.7±79.7 km for 12 females, *P*<0.01). The migrating distance was significantly correlated to the body size (*r* ^2^ = 0.37, *P*<0.05).

## Discussion

Hermaphroditism is quite common in fish, being sequential in the most cases [62]. Sex reversal has evolved independently in more than 350 species from at least 23 teleost families [63] whereas our understanding of the mechanisms underlying this phenomenon is not comprehensive [63]. The Asian seabass is a protandrous hermaphroditic species. In its early life, all individuals are male but when they attain certain body size/weight or age, the majority of them become female [32], [33]. In this study, we found that the sex ratio for Asian seabass at the age of 3–4 years was female-biased (F:M  = 1.36) in all four sampling locations in South China Sea. At the same age, the females were heavier and longer than the males. Similar results have been seen in adult brooders of Asian seabass in breeding populations at the age of 3–4 years (Mr Tan J. personal communication). In Australia, *L. calcarifer* had a size-related sex ratio [30], [32]. The smaller length classes were almost exclusively male, and the percentage of females increasing with increased total length [32]. Currently, there are several theories that can explain the sex reversal in fishes [64], [65], [66], [67]. In most fish species, female fecundity increases dramatically with body size whereas even small mature males can produce a large number of sperm to fertilize a lot of eggs [68]. Thus, selection pressures may favour the sex change from male to female. However, female to male sex reversal is more prevalent in fishes [69]. Therefore, the evolutionary mechanisms underlying male to female sex reversal in fish merit further investigation.

Both the *F_ST_* analysis and AMOVA showed that genetic differentiation of *L. calcarifer* among the four sampling locations were statistically significant, although the pairwise *F*_ST_ values were small. In Australia, the populations of *L. calcarifer* also showed significant differentiation among and within locations [35], [42], [70], [71]. The reasons for significant differentiation of *L. calcarifer* in Australia were the discontinuous distribution of appropriate habitats, an absence of extensive pre-spawning migrations and limited dispersal opportunity due to physical barriers [70], [71], [72]. While in Southeast Asia, along the cost of the South China Sea, there is no obvious physical barrier to prevent the migration of fish. In this study, detailed clustering analysis of all 549 individuals using a Bayesian based method [56] revealed that the 549 individuals collected in four locations likely formed 15 subpopulations. These data suggest that the significant population differentiation is related to not only geographical locations and but also the biological characters of the Asian seabass. Previous tagging and recapture studies showed that juvenile *L. calcarifer* remained resident until sexual maturity at the age of 3–4 years [37], [38], and some adult individuals of *L. calcarifer* were resident while others migrated [36], [37], [38], [39]. However, these previous studies did not examine which sex of individuals migrated more. In this study, the clustering analysis based on a Bayesian method clearly demonstrated that more females migrated than males in all four sampling locations. This result suggests that the different disposal of the two sexes may contribute to the population differentiation of *L. calcarifer* in the South China Sea.

Few studies have examined sex-biased dispersal patterns in marine fish species. Female-biased dispersal has been reported previously in a few species such as migratory salmonids [23], [24], [73]. To our best knowledge, our study is the first study on sex-biased dispersal in a protandrous hermaphroditic marine fish species. In this study, the differences in relatedness (r) and population divergence indices (*F*_ST_) estimated using software FSTAT v.2.9.3 were significantly (*P*<0.01) higher for males than for females in Asian seabass. Furthermore, the results from the comparisons of mean assignment index (mAIC, *P*<0.01) indicate female-biased dispersal. Although three (i.e. *F*_ST,_ *r* and mAIC) of the four genetic parameters estimated using software FSTAT v.2.9.3 [51], provided genetic evidence for female-biased dispersal in Asian seabass, the parameter *F_IS_* did not support female-biased dispersal. According to Goudet et al. [59], among the four parameters estimated by software FSTAT v.2.9.3, *F*_IS_ showed the lowest power in detecting sex-biased dispersal [51], which may explain why the parameter *F_IS_* did not support female-biased dispersal. Furthermore, the clustering analysis based on a Bayesian method clearly demonstrated that more females migrated than males in all four sampling locations. Therefore, our molecular data provide clear indirect genetic evidence for female-biased dispersal in Asian seabass. While these results may seem to be straightforward to support female-biased dispersal, it is also possible that protandrous hermaphroditism in itself generated the observed results of lower differentiation among females than males. If males dispersed before sex change, and males and females dispersed equally, then migrant males would be recruited into the female pool, but there was no recruitment in the opposite direction. Hence, the results may not represent female-biased dispersal but only a feature of the peculiar phenomenon of sex-reversal. However, this possibility can be ruled out, as previous tagging and recapture studies showed that juvenile *L. calcarifer* remained resident until sexual maturity (i.e. until starting sex change) at the age of 3–4 years [37], and during the breeding season, sexually matured individuals migrated to spawning grounds for breeding [35], [36], [38]. We analyzed the records of long term of tagging and recapture experiments on *L. calcarifer* conducted by Australian scientists in past years. We found that females moved longer distance than males. In 2011, an escape of cultured adult Asian seabass happened in Singapore. Several months after the escape, in a place 20 Km away from the escaping places, fishmen captured large females containing electronic tags, but no males (http://www.topix.com/forum/city/sweetwater-tn/TKL7F0RQR2GADD7U8), which may indicate that females moved more than males. Altogether, these data provide strong evidences for female-biased dispersal in Asian seabass, which contradicts our initial hypothesis that in *L. calcarifer*, dispersal of males and females is unbiased.

Inbreeding avoidance is regarded as one of the most important causes of sex-biased dispersal in birds and mammals [3], [14], [74]. Theory predicts that the sex that suffers from the highest disadvantage of inbreeding should be the dispersing sex [75]. In polygynous and promiscuous mating systems, inbreeding costs are different for males and females. Females invest more on reproduction in these mating systems in comparison to males. By siring a relative’s offspring, males do not lose other mating opportunities in polygynous and promiscuous systems, whereas an inbred offspring would rather replace a potentially outbred descendant in females. Inbreeding is much more costly for females than males. Therefore, females should actually be the dispersing sex in polygynous systems (Waser et al. 1986). Sex-biased dispersal is usually sufficient to minimize inbreeding [3], [76], [77]. In Asian seabass, for males, mating with relatives does not necessarily imply a cost as males can mate repeatedly all year around. The opposite is true for females as they typically reproduce once every three months in Southeast Asia [34]. This asymmetry of costs between the sexes could lead to the evolution of female-biased dispersal in Asian seabass. In addition, the female-biased sex ratio might make it difficult for females to find suitable males for mating to improve their reproductive success if they do not migrate to other places. On the other hand, for the males, mate competition is low in Asian seabass, as at the age of 3–4 years, the sex ratio was female-biased. Therefore, a female-biased dispersal pattern in Asian seabass may also be associated with female mate choice under the condition of low mate competition among males.

According to the resource competition theory (Greenwood 1980), the residing sex is the sex that takes most advantages by staying at its birth site. If males are responsible for defending the breeding territory significantly, then the benefits of philopatry will be greater for males than for females. In Asian seabass, males may enhance their own fitness by defending a territory that can attract large females for mating and provides resources for a large number of offspring. Asian seabass individuals are mostly solitary and may defend territories near submerged structures that they use as hiding spots [30]. Although currently we do not know exactly which sex acquires and defends a breeding territory, there are some indications that males may defend breeding territories (Mr. Yeo, personal communication). Therefore, it is possible that the female-biased dispersal of Asian seabass is also related to the resource competition among males.

In this study, analyzing the tagging-recapture data released by Australian researchers revealed that the migrating distance was significantly related to the body size in adult *L. calcarifer*. It is generally believed that female-biased dispersal is a cause of female-biased sexual size dimorphisms in fish species [25], [27], [78], [79], as the longer movement of females increases the feeding success, resulting in rapid growth as compared with males. For example, in sablefish females grew larger and moved further away than males [27]. However, in Asian seabass, this may not be the case. Under aquaculture conditions, enough feed was usually given to the young fishes, and at the same age (3–4 years) females usually grow bigger than males. Therefore, feeding resources could not be a determining factor promoting female-biased size dimorphisms in Asian seabass. It is highly possible that at the same age, in wild populations, the bigger size of females of Asian seabass makes them migrate faster than males, which in turn gives females more chances to find and mate suitable males in the populations where the sex ratio is female-biased. Therefore, the female-biased sexual size dimorphisms may be a cause of female-biased movements.

### Conclusions

Our genetic data, in combination with data of studies on tagging and recapture published by Australian scientists in the past years [35], [37], [38], [80], provided strong evidences for female-biased gene flow among populations of Asian seabass. We suggest that inbreeding avoidance and female mate choice under the condition of low mate competition among males, as well as male resource competition created a female-biased dispersal. The female-biased sexual size dimorphisms may be a cause of the female-biased movements. The ultimate causes behind sex reversal and sex-biased dispersal and the potential evolutionary implications of female-biased dispersal in protandrous hermaphroditic marine fish species warrant further investigation. Studies using DNA markers, in combination with data from tagging-recapture studies in the hermaphroditic fish species, could enhance our understanding of the dispersal of fish.
